# Supplementary material for: Enhancing the Photocatalytic Activity of Halide Perovskite Cesium Bismuth Bromide/Hydrogen Titanate Heterostructures for Benzyl Alcohol Oxidation
Source: Nanomaterials (Basel). 2024 Apr 25;14(9):752. doi: 10.3390/nano14090752 (PMC11085227; doi:10.3390/nano14090752)
Supplement: Supplementary file 1 [file nanomaterials-14-00752-s001.zip › nanomaterials-2939996-supplementary.pdf]

nanomaterials

Article

Enhancing the Photocatalytic Activity of Halide Perovskite Cesium Bismuth Bromide/Hydrogen Titanate Heterostructures for Benzyl Alcohol Oxidation

Huzaikha Awang [1,2], Abdo Hezam [3], Tim Peppel [1,*] and Jennifer Strunk [1,3,*]

1 Leibniz Institute for Catalysis, Albert-Einstein-Str. 29a, 18059 Rostock, Germany; huzaikha.awang@catalysis.de
2 Preparatory Centre for Science and Technology, Universiti Malaysia Sabah, Jalan UMS, Kota Kinabalu 88400, Sabah, Malaysia
3 School of Natural Sciences, Technical University of Munich (TUM), Lichtenbergstr. 4, 85748 Garching, Germany; abdo.mohsen@tum.de
* Correspondence: tim.peppel@catalysis.de (T.P.); jennifer.strunk@tum.de (J.S.)

Table S1. Comparison of photocatalysts for oxidation of BnOH to BzH and BzA.

Materials	Condition	Conversion (%)	Selectivity (%) / Conversion Rate of BzH	Selectivity (%) / Conversion Rate of BzA	Reference
CBHTNS-30	2 h<br>O_2<br>Blue Light<br>20 mM<br>10 mg<br>2 ml	98	21 / 1.2 mmol g^{-1} h^{-1}	75 / 3.7 mmol g^{-1} h^{-1}	This Work
$Cs_3Bi_2Br_9/TiO_2$	4 h<br>Air<br>0.1 mM<br>10 mg<br>3 ml	58.6	> 99 / 1.5 mmol g^{-1} h^{-1}	-	[1]
$CsPbBr_3/TiO_2$	20 h<br>O_2<br>Vis. Light<br>0.1 M<br>25 mg<br>5 ml	40	99/-	-	[2]
Cs_2TeBr_6	2 h<br>UV	-	- / 16.7 µmol	- / 1.5 µmol	[3]
$Cs_3Bi_2Br_9$	2 h<br>LED	-	- / < 6.0 µmol	-	
TiO_2/Ti_3C_2	5 h<br>O_2<br>Vis. Light<br>20 µmol<br>30 mg<br>25 ml	97	98 / -	-	[4]
Au–Pd/ $H_2Ti_3O_7$	6 h<br>O_2	91	72.6 / 139 mol/kg.h	18.5 / -	[5]

Au$_1$Pt$_1$/TiO$_2$	halogen light<br>0.1 g<br>10 ml<br>8 h<br>O$_2$	65.3	> 98 / -	-	[6]
0.5 wt% Au-0.5 wt% Pd/TiO$_2$	Vis. Light<br>0.05 M<br>50 mg<br>20 ml<br>4 h<br>TBHP as oxidant agent	19	> 80.5 /-	-	[7]

Table S2. Some of the recent literature on photodegradation performance of halide perovskite and hydrogen titanate.

Materials	Pollutant	Condition	Efficiency (%)	Reference
$Cs_3Bi_2I_9$ perovskite	Organic dyes (RhB)	3 h, visible light	93	[8]
$Cs_3Bi_2Br_9$ perovskite	Organic dyes (MB)	1.5 h, Visible light	80	[9]
$Cs_3Bi_2Br_9/TiO_2$ heterojunction	2-mercaptobenzothiazole (MBT)	20 min, visible light	99.9	[1]
$C_3N_4/Cs_3Bi_2Br_9$ composite	Organic dyes (RhB)	1 h, solar simulator	98	[10]
2D tri-titanate ($H_2Ti_3O_7$) nanosheet	Organic dyes (MB and RhB)	10 h, UV light	99.9 and 92.7	[11]
2D tri-titanate ($H_2Ti_3O_7$) nanosheets (TNS) and nanotubes (TNT)	Tar and Nicotine of Cigarette smoke (CS).	24 h using machine-smoked	Tar<br>TNS : 52%<br>TNT : 70%<br><br>Nicotine<br>TNS : 50%<br>TNT : 67%	[12]

References

1. Sun, Q.; Ye, W.; Wei, J.; Li, L.; Wang, J.; He, J.-H.; Lu, J.-M. Lead-free perovskite $Cs_3Bi_2Br_9$ heterojunctions for highly efficient and selective photocatalysis under mild conditions. *Journal of Alloys and Compounds* **2022**, *893*, 162326, doi:https://doi.org/10.1016/j.jallcom.2021.162326.
2. Schünemann, S.; van Gastel, M.; Tüysüz, H. A $CsPbBr_3/TiO_2$ Composite for Visible-Light-Driven Photocatalytic Benzyl Alcohol Oxidation. *ChemSusChem* **2018**, *11*, 2057-2061, doi:https://doi.org/10.1002/cssc.201800679.
3. Jin, J.; Huang, H.; Chen, C.; Smith, P.W.; Folgueras, M.C.; Yu, S.; Zhang, Y.; Chen, P.-C.; Seeler, F.; Schaefer, B.; et al. Benzyl Alcohol Photo-oxidation Based on Molecular Electronic Transitions in Metal Halide Perovskites. *ACS Photonics* **2023**, *10*, 772-779, doi:https://doi.org/10.1021/acsphotonics.3c00042.

4. Bao, X.; Li, H.; Wang, Z.; Tong, F.; Liu, M.; Zheng, Z.; Wang, P.; Cheng, H.; Liu, Y.; Dai, Y.; et al. TiO_2/Ti_3C_2 as an efficient photocatalyst for selective oxidation of benzyl alcohol to benzaldehyde. *Applied Catalysis B: Environmental* **2021**, *286*, 119885, doi:https://doi.org/10.1016/j.apcatb.2021.119885.
5. Du, M.; Zeng, G.; Huang, J.; Sun, D.; Li, Q.; Wang, G.; Li, X. Green Photocatalytic Oxidation of Benzyl Alcohol over Noble-Metal-Modified $H_2Ti_3O_7$ Nanowires. *ACS Sustainable Chemistry & Engineering* **2019**, *7*, 9717-9726, doi:https://doi.org/10.1021/acssuschemeng.9b01817.
6. Zhang, X.-F.; Wang, Z.; Zhong, Y.; Qiu, J.; Zhang, X.; Gao, Y.; Gu, X.; Yao, J. TiO_2 nanorods loaded with AuPt alloy nanoparticles for the photocatalytic oxidation of benzyl alcohol. *Journal of Physics and Chemistry of Solids* **2019**, *126*, 27-32, doi:https://doi.org/10.1016/j.jpcs.2018.10.026.
7. JamJam, N.M.; Taufiq Yap, Y.H.; Muhamad, E.N.; Izham Saiman, M.; Saleh, T.A. Free solvent oxidation of molecular benzyl alcohol by newly synthesized AuPd/titania catalysts. *Inorganic Chemistry Communications* **2019**, *107*, 107471, doi:https://doi.org/10.1016/j.inoche.2019.107471.
8. Bresolin, B.-M.; Günnemann, C.; Bahnemann, D.W.; Sillanpää, M. Pb-Free $Cs_3Bi_2I_9$ Perovskite as a Visible-Light-Active Photocatalyst for Organic Pollutant Degradation. *Nanomaterials* **2020**, *10*, 763, doi:https://doi.org/10.3390/nano10040763.
9. Masri, M.; Girisha, K.B.; Hezam, A.; Qahtan, T.F.; Alkanad, K.; Masri, F.; Namratha, K.; Udayabhanu; Byrappa, K. Enhanced photocatalytic activity and stability of 2D Cs3Bi2Br9 perovskite nanosheets synthesized via modified antisolvent method. *Colloids and Surfaces C: Environmental Aspects* **2024**, *2*, doi:https://doi.org/10.1016/j.colsuc.2023.100024.
10. Xu, W.-L.; Hu, J.; Yang, Q.; Lian, Y.; Zheng, M.; Du, E. Charge transfer dynamics in C3N4 encapsulated Cs3Bi2Br9 nanocrystals heterojunction for photocatalytic application. *Journal of Alloys and Compounds* **2024**, *988*, doi:https://doi.org/10.1016/j.jallcom.2024.174275.
11. Hua, S.; Yu, X.; Li, F.; Duan, J.; Ji, H.; Liu, W. Hydrogen titanate nanosheets with both adsorptive and photocatalytic properties used for organic dyes removal. *Colloids and Surfaces A: Physicochemical and Engineering Aspects* **2017**, *516*, 211-218, doi:https://doi.org/10.1016/j.colsurfa.2016.12.031.
12. Deng, Q.; Huang, C.; Xie, W.; Zhang, J.; Zhao, Y.; Hong, Z.; Pang, A.; Wei, M. Significant reduction of harmful compounds in tobacco smoke by the use of titanate nanosheets and nanotubes. *Chemical Communications* **2011**, *47*, 6153-6155, doi:https://doi.org/10.1039/c1cc10794a.
